# Immunological Basis for the Gender Differences in Murine *Paracoccidioides brasiliensis* Infection

**DOI:** 10.1371/journal.pone.0010757

**Published:** 2010-05-21

**Authors:** Camila Figueiredo Pinzan, Luciana Pereira Ruas, Anália Sulamita Casabona-Fortunato, Fernanda Caroline Carvalho, Maria-Cristina Roque-Barreira

**Affiliations:** Departamento de Biologia Celular e Molecular e Bioagentes Patogênicos, Universidade de São Paulo, Ribeirão Preto, São Paulo, Brasil; University of California Los Angeles, United States of America

## Abstract

This study aimed to investigate the immunological mechanisms involved in the gender distinct incidence of paracoccidioidomycosis (pcm), an endemic systemic mycosis in Latin America, which is at least 10 times more frequent in men than in women. Then, we compared the immune response of male and female mice to *Paracoccidioides brasiliensis* infection, as well as the influence in the gender differences exerted by paracoccin, a *P. brasiliensis* component with carbohydrate recognition property. High production of Th1 cytokines and T-bet expression have been detected in the paracoccin stimulated cultures of spleen cells from infected female mice. In contrast, in similar experimental conditions, cells from infected males produced higher levels of the Th2 cytokines and expressed GATA-3. Macrophages from male and female mice when stimulated with paracoccin displayed similar phagocytic capability, while fungicidal activity was two times more efficiently performed by macrophages from female mice, a fact that was associated with 50% higher levels of nitric oxide production. In order to evaluate the role of sexual hormones in the observed gender distinction, we have utilized mice that have been submitted to gonadectomy followed by inverse hormonal reconstitution. Spleen cells derived from castrated males reconstituted with estradiol have produced higher levels of IFN-γ (1291±15 pg/mL) and lower levels of IL-10 (494±38 pg/mL), than normal male in response to paracoccin stimulus. In contrast, spleen cells from castrated female mice that had been treated with testosterone produced more IL-10 (1284±36 pg/mL) and less IFN-γ (587±14 pg/mL) than cells from normal female. In conclusion, our results reveal that the sexual hormones had a profound effect on the biology of immune cells, and estradiol favours protective responses to *P. brasiliensis* infection. In addition, fungal components, such as paracoccin, may provide additional support to the gender dimorphic immunity that marks *P. brasiliensis* infection.

## Introduction

Paracoccidioidomycosis (PCM) is the most important endemic deep mycosis in Latin America. The disease is caused by the dimorphic fungus *Paracoccidioides brasiliensis* (Pb)[1], [2]. Infection is acquired by inhalation of the airborne conidia, derived from the mycelial form of the fungus, which once in the alveoli transform into the yeast infective form [1], [3]. Clinical forms of the disease range from asymptomatic pulmonary lesions to systemic generalized infections. The severity of PCM depends on the balance between the virulence of the pathogen and the host responses [2], [4], [5].

The clinical incidence and progression of paracoccidioidomycosis in endemic areas is greatly higher in men than in women, in a ratio reported to be as huge as 13:1 [1], [2], [4], [6]. The similar detection of subclinical infection in healthy individuals, by delayed-type hypersensitivity to paracoccidioidin, reveals that both sexes acquire infection at the same rate [7], whereas the progression to overt disease is much more frequent in males. These epidemiologic data have supported the hypothesis that hormonal factors play a critical role in the pathogenesis of PCM. *In vitro* experiments have demonstrated that the female hormone 17β-estradiol inhibits the transformation of both mycelial and conidia forms into yeast cells [8], [9]. Moreover, 17β-estradiol, but not testosterone, notably reduces the growth of yeast cells. These in vitro observations are evidences for the direct role exerted by estrogens in the fungus [10]. Consistently, *in vivo* studies have shown that female mice, especially at estrus, achieve a much higher clearance of the yeast cells than male mice [10]. In a 96 h period after conidia inoculation, transition to yeast is identified in the lung of male mice, but not in female mice [11]. The infection of mice castrated and inversely hormone reconstituted showed that castrated male mice reconstituted with 17β-estradiol rapidly restrict fungal proliferation, whereas castrated female mice reconstituted with testosterone are unable to restrict the disease [12]. In spite of the clear demonstration of a pronounced sex bias in *P. brasiliensis* infection, only the hormonal effect directly exerted on the fungus has been examined, whereas no investigation on the immunological contribution for the sexual differences observed during paracoccidioidomycosis has been carried out.

Clinical and experimental studies have established that cell-mediated immune response plays a crucial role in host defence against *P. brasiliensis* infection [13], [14], [15]. In the asymptomatic form of pcm, T-helper type 1 (Th1) specific immune response occurs, while Th2 immunity is associated with severe disease [16], [17], [18]. These immunity patterns governing resistance or susceptibility to the fungus have been extensively demonstrated in murine models of infection, especially those comparing the course of the mycosis in A/Sn and B/10.A inbred mice, which mimic the polarized clinical forms of the human pcm [19]. By using appropriate knockout mice, the role of cytokines, mainly the one exerted by IL-12, IFN-γ and TNF-α, in the protective immunity to *P .brasiliensis* infection has been clearly proven [20], [21], [22]. The mechanisms of fungal killing by cytokines-activated macrophages have also been recognized [23], [24], [25].

We have previously demonstrated that *P.brasiliensis* yeasts release and express a GlcNAc-binding lectin designated paracoccin on their surface [26], [27]. Besides interacting in a sugar-recognition dependent manner with laminin, paracoccin stimulates macrophages to produce high levels of TNF-α and nitric oxide, which are involved in the fungicidal activity of these cells.

The aim of the present study was to compare the immune response triggered by *P. brasiliensis* infection in male and female mice, with emphasis on the role exerted by paracoccin in the hypothetical gender differences.

## Results

### Spleen cells from male and female infected mice secrete different levels of cytokines following paracoccin stimulus

**Figure 1:**
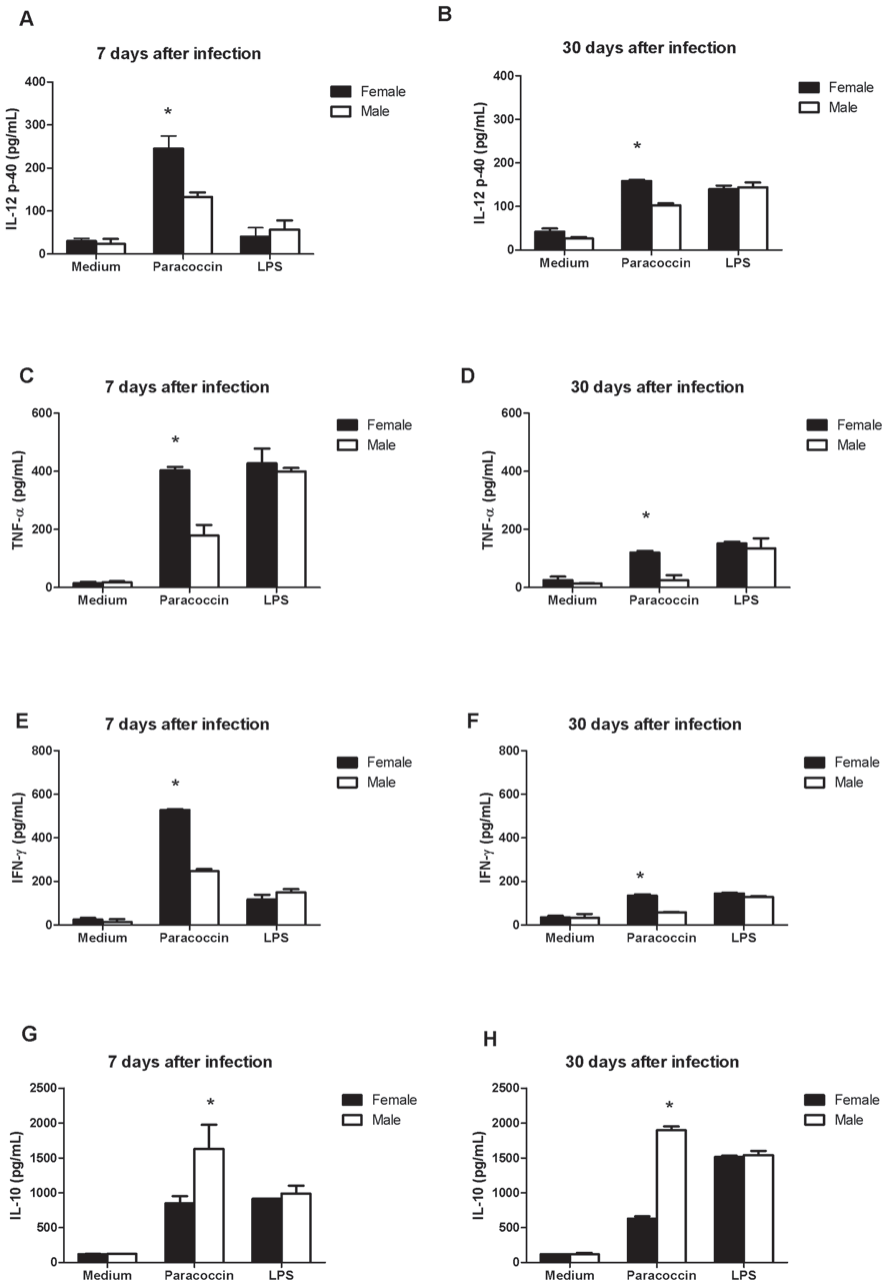
Spleen cells from male and female infected mice secrete different levels of cytokines after paracoccin stimulus. Spleen cells of mice of both sexes (n = 6) infected with *P.brasiliensis* yeast were collected after 7 and 30 days post infection and cultured for 48 hours under stimulation with paracoccin (0.7 µg/ml), LPS (1 µg/ml) or only medium. The concentrations of IL-12p40, TNF-α, IFN-γ and IL-10 in supernatants were measured by ELISA. Each bar represents the mean ± SD of duplicates and is representative of four experiments made in duplicate. * p<0.05 significant female versus male of the same group. All cytokine data were normalized against those of the non-infected male and female mice.

Considering that males display higher incidence of human pcm and higher severity of experimental pcm [2], [4], [11], [12], we have investigated whether an immunological mechanism could account for this gender difference. Spleen cells from male and female mice were collected 7 and 30 days after inoculation of *P. brasiliensis* yeasts and cultivated for 48 hours under stimulus with paracoccin, LPS, or medium only. Assessment of Th1 cytokines production by the spleen cells showed that at day 7 post-infection, the levels of IL-12p40 (134±64 pg/mL), TNF-α (402±25 pg/mL), and IFN-γ (527±9 pg/mL) produced by cells from female mice stimulated with paracoccin were significantly (p<0.05) higher than those produced by cells from male mice (132±15 pg/mL, 179±71 pg/mL, and 247±20 pg/mL, respectively (Figure 1A, C, E). As expected, lower cytokines levels were detected in the supernatants of spleen cells from both male and female mice at day 30 post-infection, compared with day 7. Even so, compared with males, spleen cells from females produced significantly higher levels of Th1 cytokines (Figure 1B, D, F). Concerning Th2 cytokines, there was a remarkable difference in IL-10 production between paracoccin-stimulated spleen cells from infected male and female mice. Spleen cells from males produced higher levels of IL-10 than those from females, resulting in a relation of two- and threefold at days 7 and 30 after infection, respectively (Figure 1G, H). IL-4 was not detected in the spleen cells supernatants (data not shown). LPS-stimulation of spleen cells also induced cytokines production, but similar levels were detected in the supernatants of cells from males and females. Notably, lower levels of IL-12 and IFN-γ were stimulated by LPS than by paracoccin in the cells collected at day 7 after infection; such differences were more pronounced for cells from females. Equivalent levels of cytokines were measured in the supernatant of unstimulated spleen cells from males and females, collected at days 7 or 30 after infection.

### Quantification of IL-4, GATA-3, T-bet and fungal burden in organs from male and female infected mice

**Figure 2:**
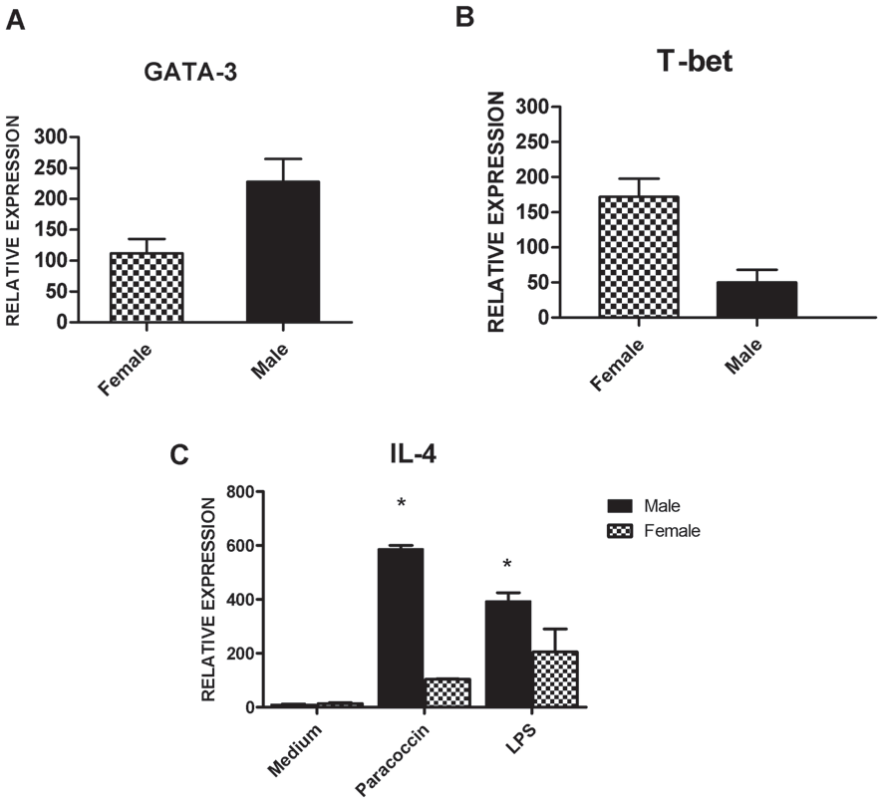
Expression of mRNA for IL-4, GATA-3 and T-bet from male and female infected mice. Spleen cells of male and female mice infected with *P.brasiliensis* yeast were collected after 30 days post infection and cultured for 48 hours under paracoccin stimulation (0.7 µg/ml) (2A, B and C), LPS (1 µg/ml) (2C) or only medium (2C). The levels of mRNA relative expression for the IL-4, T-bet and GATA-3 were determined by real-time PCR, using the β-actin gene as control. All real-time PCR data were normalized against those of the non-infected male and female mice. The results represent the mean±SD of six mice per group, from a representative experiment of three assays. * p<0.05 significant when compared with opposite sex.

**Figure 3:**
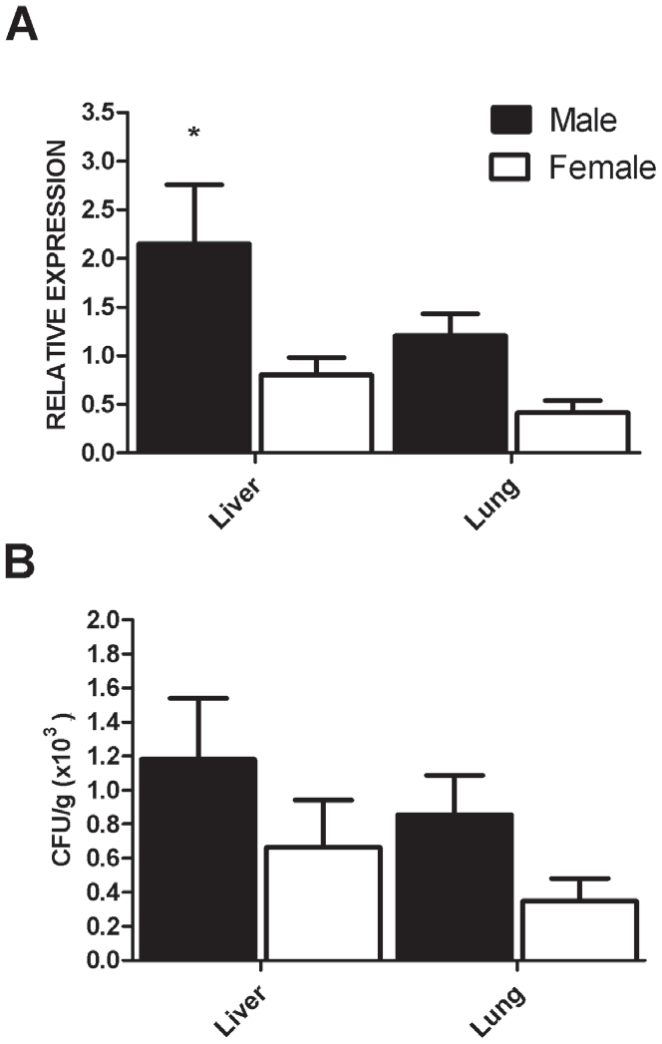
Quantification of *P. brasiliensis* through real-time PCR and Colony-forming units (CFU). Evaluation of the fungal burdens in lungs and liver of male (grey bars) and female (open bars) mice 30 days after intraperitoneal infection with yeast cells. The bars represent the mean±SD obtained from duplicate samples in groups of six animals. * P<0.05, compared with female infected mice.

Because susceptibility to PCM is associated with the occurrence of type 2 immunity (Th2) and IL-4 was not detectable in the spleen cells supernatants, we next measured the mRNA levels of this cytokine, as well as the expression levels of GATA-3 and T-bet transcription factors, respectively associated with Th2 and Th1 cell differentiation. The expression of IL-4 mRNA was 82% higher in paracoccin-activated spleen cells from male compared with those from female mice, collected at day 30 post-infection. A significant disparity on IL-4 mRNA expression was also observed for LPS-stimulated spleen cells from males and females (Figure 2C). A higher relative expression of the T cell transcription factor GATA-3 was stimulated by paracoccin in cells from males, whereas T-bet expression largely predominated in cells from females (Figure 2A and B). Our results suggest that a stronger Th2 immune response is developed in male mice, whereas female mice develop a Th1 biased immunity. The quantitative real-time PCR specific for *P.brasiliensis* mRNA and the quantification of colony-forming units (CFU) show that the fungal burden on liver and lungs of male mice was dramatically higher than that in female mice (Figure 3A and B). This result suggests that the hormonal status might acts controlling the clearance of *P. brasiliensis* yeasts.

### Macrophages from female mice have a more efficient fungicidal ability than those from male mice

**Figure 4:**
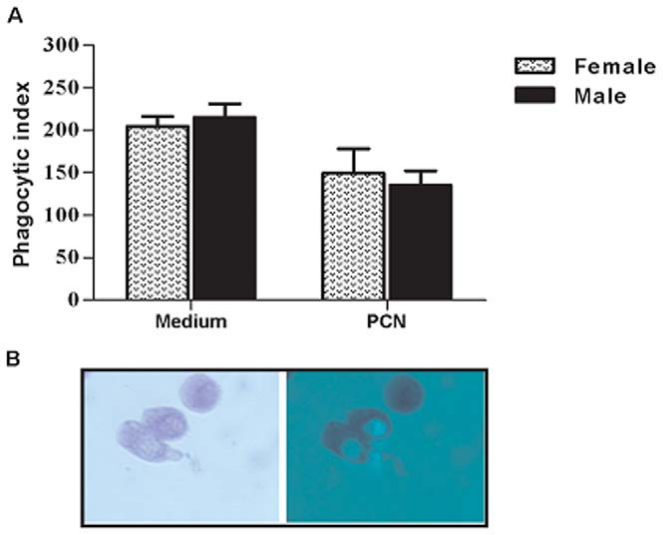
Phagocytosis of *P. brasiliensis* yeasts by macrophages from male and female mice. Macrophages treated or not with paracoccin (50 µg/well) were challenged with Pb *FITC*- conjugated yeasts for 4 hs. The cells were stained with Evans Blue and counted in a fluorescence microscope (100x) to determine the yeast Phagocitic index (3A). Panel B illustrates the result and allow to observe that no extracellular attachment of yeasts after 4 hs of incubation. The results on panel A represent the mean±SD from a representative experiment of three assays.

Resistance against P. *brasiliensis* infection is critically dependent on activated macrophages, which exert phagocytic and fungicidal roles. Such activities are modulated by fungal components and host factors, hormones included [28]. Therefore, the abilities of male and female macrophages to phagocyte and kill *P. brasiliensis* yeasts were compared. The effect of paracoccin-stimulation on these macrophage abilities was also evaluated. Macrophages from male and female mice were similarly able to phagocyte *P. brasiliensis* yeasts. Although phagocytosis was inhibited when paracoccin was added to the culture medium (Figure 4A), the phagocytic index showed by macrophages from females and males were still similar. Internalization of Pb *FITC*- conjugated yeasts was observed by fluorescence microscopy, as illustrated in Figure 3B, after 4-hour incubation with mice peritoneal macrophages. No extracellular attachment of yeasts was observed.

**Figure 5:**
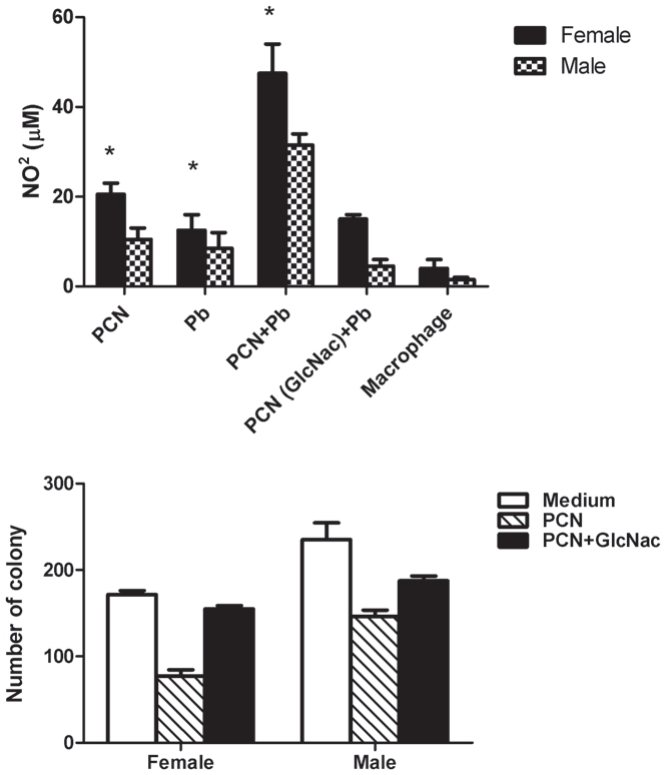
NO production and viability of Pb yeasts after phagocytosis by macrophages from male and female mice. Macrophages from male and female mice were incubated with paracoccin (PCN), *P. brasiliensis* yeasts (Pb), PCN plus Pb, PCN (pre-treated with GlcNac) plus Pb or only medium and nitrite levels were detected by the Griess method (4A). For the evaluation of viability of Pb cells macrophages were stimulated with PCN, PCN (pre-treated with GlcNac) or only medium and challenged with live Pb. The determination of the number of viable fungi was made by CFU counts (4B). The results represent the mean±SD from a representative experiment of three assays. * p<0.05 significant when compared with male of the same group.

We have previously reported that paracoccin stimulates NO release by macrophages [27]. Considering that NO provides macrophages with an effector mechanism to kill *P. brasiliensis* yeasts, in this work we have measured NO release by elicited macrophages from males and females. The macrophages stimulus with paracoccin (PCN) or yeasts (Pb) triggered moderate elevation of nitrite production (Figure 5A). The highest NO production was achieved with the association of paracoccin and yeast, which provided nitrite levels twice as high as those induced by paracoccin alone. Previous incubation of paracoccin with the monosaccharide GlcNAc inhibited the lectin property of inducing NO production, since the nitrite levels decreased below 15 µM. Higher levels of nitrite were provided by macrophages from female mice compared with male mice, in all assayed conditions. To evaluate the efficacy of the macrophage fungicidal activity, we quantified the yeasts viability when co-cultured with macrophages from males or females, in the presence or absence of paracoccin. The significantly lower recovery of fungal CFU from co-cultures with macrophages from females (Figure 5B) indicates that these cells eliminate the *P. brasiliensis* yeasts more efficiently, especially under stimulation with paracoccin. Although paracoccin increases the fungicidal ability of macrophages obtained from both sexes, the number of CFU dropped by 54% when co-cultures were performed with macrophages of females and by 37% in co-cultures with macrophages from males. The paracoccin effect of enhancing yeast killing was blocked by lectin pre-incubation with GlcNAc, in a demonstration that such activity is dependent on the paracoccin sugar-recognition property.

### Sex hormones modulate the cytokines production of spleen cells from male and female infected mice

**Figure 6:**
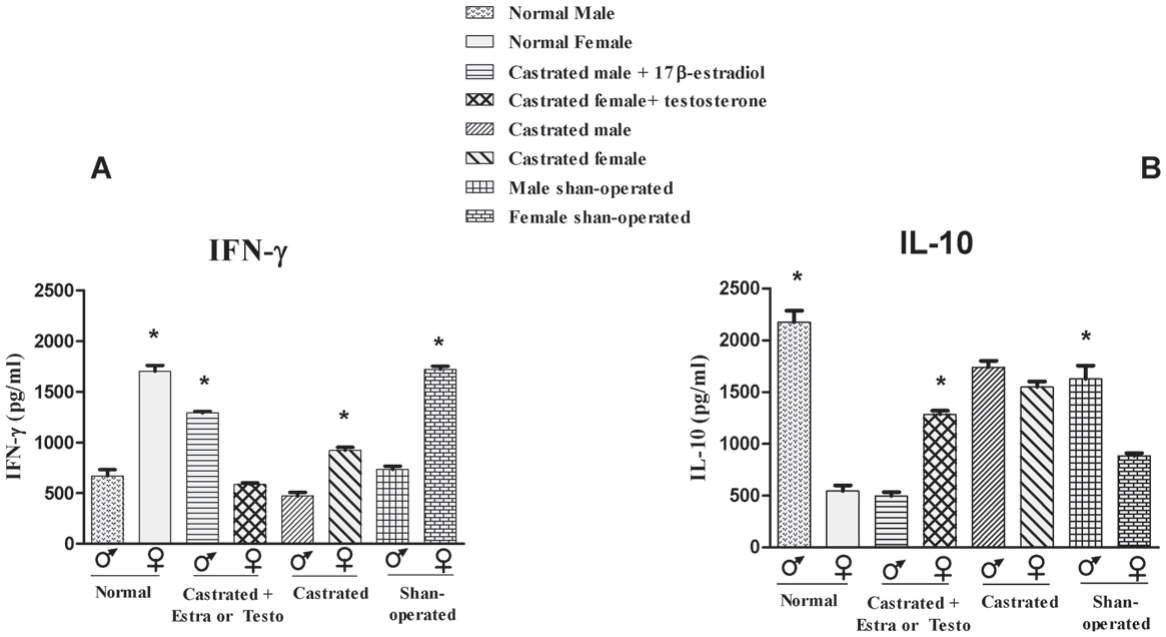
IFN-γ (A) and IL-10 (B) production by spleen cells from male and female infected mice of different treated groups. Spleen cells from male and female 30 days infected mice from different groups were collected and cultured for 48 hours under paracoccin stimulation (7 µg/ml). The concentration of IFN-γ and IL-10 in supernatants was measured by ELISA. The results represent the mean±SD of six mice per group, from a representative experiment of two assays. * p<0.05 significant when compared with opposite sex of the same group.

Since we found remarkable differences in the behaviour of male and female cells, especially following stimulation with paracoccin, the role of sexual hormones was directly examined. For this purpose, we performed gonadectomy followed by inversely hormone reconstitution. Animals were placed in eight experimental groups: intact female, intact male, castrated females, castrated males, castrated females treated with testosterone, castrated males treated with estradiol, and Shan-operated females and males. Spleen cells collected 30 days after *P.brasiliensis* inoculation were cultivated for 48 hours under paracoccin stimulus. The collected supernatants were examined in terms of IFN-γ and IL-10 levels. As expected, on the basis of the results shown in Figure 1, IFN-γ production by spleen cells of intact female mice was twofold higher than the production by cells from intact male mice (Figure 6A). Castration was associated with a decline in IFN-γ production by spleen cells from mice of both sexes; such an effect was not observed with cells from Shan-operated mice. Cells from castrated male mice, to which estradiol was given, produced IFN-γ in higher concentrations (1291 pg/mL) compared with cells from intact or castrated male mice (670 and 470 pg/mL, respectively). In contrast, cells from castrated female mice, reconstituted with testosterone, presented decreased IFN-γ production (587 pg/mL) compared with cells from intact or castrated female mice (1707 and 921 pg/mL, respectively). IL-10 production, in consonance with the results shown in Figure 1, was higher by spleen cells from intact male mice (2171 pg/mL) than by cells from intact females (542 pg/mL) (Figure 6B). Female mice castration was associated with a threefold rise in IL-10 production (1579 pg/mL), and levels close to the ones provided by cells from the Shan-operated male mice (1627 pg/mL) were attained. Cells from castrated male mice inversely reconstituted with estradiol decreased IL-10 production by over 77% compared with cells from intact male mice. On the other hand, castrated female mice treated with testosterone increased IL-10 production by 57% compared with cells from intact female mice (Figure 6B). Cells obtained from Shan-operated male and female mice produced IFN-γ and Il-10 levels that were not significantly different from those provided by the cells from male and female intact mice (Figures 6A and 6B). These results demonstrate that the hormonal milieu influences cytokine production by cells from *P. brasiliensis* infected mice, a fact that may account for the distinct susceptibility of male and female mice to paracoccidioidomycosis.

## Discussion

The carefully orchestrated events that result in protective immunity against infections are coordinated by a regulated communication between the endocrine and immune systems. The immune response is considered to be sexually dimorphic, both in type and magnitude. In general, following infection, females develop more effective and potentially protective humoral and cell-mediated responses, while males show higher frequency and severity of several fungal, viral, bacterial, and parasitic diseases [29], [30], [31]. Such a gender difference is definite in paracoccidioidomycosys, whose higher incidence and gravity in males compared with females are detectable among both patients [32], [33] and experimentally infected animals [11]. This picture has been attributed to the effect exerted by estrogen on the fungus, inhibiting conidium-to-yeast conversion [8], [9] and yeast growth [10], [34], observations that were reinforced by the detection of an estrogen receptor in yeast cytosol [35].

Almost certainly, the gender differences in paracoccidioidomycosis are not only due to the hormonal effects on fungal transition and growth. The hypothesis that the host immunity would also be affected has already been mentioned [12], but never cautiously investigated. The present study contributes to clarifying some aspects of the intricate mechanisms of immunity developed by male and female mice during *P.brasiliensis* infection. Higher levels of IL-12, IFN-γ and TNF-α, cytokines that correlate with resistance to the infection [19], [36], have been produced by spleen cells from infected females, in response to paracoccin stimulus. In contrast, spleen cells from males, in similar conditions, produced higher levels of IL-10, which plays a major role in the antigen-specific immunosupression of paracoccidiodomycosis [16], [18], [37], [38]. These data are coherent with the known effects of sex hormones on the expression of cytokines. Estrogens stimulate IL-12 [39], IFN-γ [40], [41], [42] and TNF-α [43], and downregulate IL-10 [44], whereas testosterone enhances the synthesis of IL-10 [45], [46]. We also found increased expression of IL-4 and GATA-3 mRNA in the spleen cells from infected male mice, while the females spleen cells, in similar conditions, enhanced the mRNA levels of the transcriptional factor T-bet. Our observations are in agreement with recent demonstration that *in vivo* estrogen treatment primes splenic cells for Th1 differentiation in the context of increased T-bet expression [47]. This indicates that the key downstream IFN-γ mediated events, which are essential in the defence against *P.brasiliensis* infection [22], are positively regulated by estrogens. On the other hand, the higher production of IL-10 by cells of infected males associated with enhanced mRNA expression for IL-4 and GATA-3 and decreased levels of IFN-γ, IL-12, and TNF-α indicate the development of a Th2-biased immunity, which account for the low fungal clearance and the consequent susceptibility of male mice to *P.brasiliensis* infection. Interestingly, the gender distinction of cytokines production, firstly observed by using spleen cells obtained at day 7 post-infection, was still present 30 days after infection. Moreover, the distinction of immunity patterns between genders was only evident when spleen cells were stimulated with the fungal antigen paracoccin. Because paracoccin is endowed of lectin property [27], we took into consideration the possibility that the direct action of lectin on spleen cells could account for the observed phenomenon.

Macrophages, which are cell targets for the paracoccin lectin activity [27], exert a leading role in the resistance against *P. brasiliensis* infection [48], [49]. It is known that their phagocytic and microbicidal activities are modulated by both fungal antigens and host endogenous factors, hormones included [28], [50], [51], [52], [53], a fact that motivated us to investigate whether macrophages from males and females differ in terms of their ability to internalize and kill *P. brasiliensis* yeasts. After the experimental verification that macrophages from both origins had similar phagocytic abilities, we demonstrated that higher amounts of nitric oxide were produced by macrophages from females compared with males, in response to incubation with either paracoccin, yeasts or paracoccin plus yeast. Consistently with the nitric oxide role in the fungicidal activity of murine macrophages [25], CFU recovery was higher after yeasts incubation with macrophages from males, whereas macrophages from females showed greater ability to kill the fungus. Although paracoccin was able to induce macrophages from females and males to produce NO and kill yeasts, the cells from females recurrently responded better. Both responses to paracoccin were inhibited in the presence of N-acetyl glucosamine, whereas a non-relevant monosaccharide (D-galactose) exerted no effect on the paracoccin properties (data not shown), suggesting that they engage the carbohydrate-recognition domain of the molecule, as demonstrated in previous studies [27].

The higher response of macrophages from females to the paracoccin stimulus may be putatively attributed to distinct glycan expression on the surface of cells from female and male mice. Several studies have suggested that cell glycosylation can be regulated hormonally [54], [55], [56], [57]. The β-estradiol effects on the glycosylation of the plasma membrane of HeLa S3 cells have been indirectly assessed through the binding of plant lectins on the cell surface. Curiously, a significant higher binding on the treated cells was determined by the N-acetyl-D-glucosamine specific lectins, respectively obtained from *Phytolacca americana* and *Triticum vulgaris*, a fact that suggests that estradiol enhanced the glycosylation state of membrane proteins[58]. Concerning macrophages, because they express estrogen receptors [59], [60], we speculate that estrogen/receptor interaction may contribute to altering the expression of glycans on the cell surface, thereby converting macrophages into good targets for paracoccin recognition. As a consequence, high amounts of nitric oxide could be produced, as verified with macrophages from female mice in our experiments. An alternative mechanism for the augmented NO production can be provided by a regulatory effect exerted directly by estrogen, as demonstrated by several authors [28], [53], [61], [62]. Exposure of peritoneal macrophages to estrogen has been shown to increase iNOS expression and nitric oxide production [61]. Quite the opposite, testosterone inhibits expression of inducible nitric oxide synthase and NO production by murine macrophages [46], [63]. All these considerations have led us to suppose that the effects exerted by estrogens on macrophages during experimental paracoccidioidomycosis may involve a multi-event process, which includes: (a) altered expression of glycans on the host cell surface; (b) augmented interaction with a GlcNAc binding fungal lectin; (c) enhancement of nitric oxide production by macrophages. By acting synergistically in the initial phases of the *P. brasiliensis* infection, these mechanisms may account for the greater resistance of female mice to paracoccidioiodomycosis.

The commitment of both the endocrine and the immune systems has been demonstrated to be required for the gender distinct susceptibility to infections. For such a demonstration in murine models, procedures such as gonadectomy and reverse hormone replacement have been crucial [12], [64], [65]. These interventions also provided important data in our study. The castration of both female and male mice resulted in decreased IFN-γ production. On the other hand, IL-10 production by female spleen cells after castration became as high as the production by spleen cells from Shan-operated males. Since after castration the profile of cytokines produced by cells from females was no longer that associated with resistance, the importance of estrogens in determining resistance to *P.brasiliensis* infection was strongly suggested. This verification is consistent with the study of Aristizabal et al. (2002), in which castrated male and female mice infected with *P. brasiliensis* showed a lower capacity to restrict disease progression, as demonstrated by higher lung CFU recovery and failure to develop pulmonary compact granulomas.

The role exerted by sex hormone in the outcome of the infection has also been demonstrated by the reverse hormone reconstitution of the castrated mice. At day 30 after infection, the spleen cells from castrated males that were reconstituted with estradiol responded to the paracoccin stimulus by producing more IFN-γ and less IL-10, reproducing the pattern of cytokines released by cells of the intact females, under similar experimental conditions. Still in the same conditions, castrated females treated with testosterone increased IL-10 and decreased IFN-γ production, thus reproducing the pattern of cytokines released by cells of the intact males. Again, these results are consistent with those reported by Aristizabal et al. (2002) concerning the fact that, at least transitorily, estrogen administration contributed to conferring more resistance to the infection in the case of castrated males, whereas treatment of castrated females with testosterone was deleterious, a fact reflected by the increased number of inflammatory foci and loose granulomas associated with high recovery of fungal colonies in their lungs.

The effects of sex hormones have been previously demonstrated in murine experimental models of *Leishmania mexicana* [66], *Plasmodium chabaui* [67], and *Micobacterium. marinum* [68]. The data provided by the literature and by the present study, show that gender distinctions play a critical role in determining susceptibility or resistance to several pathogens.

**Figure 7:**
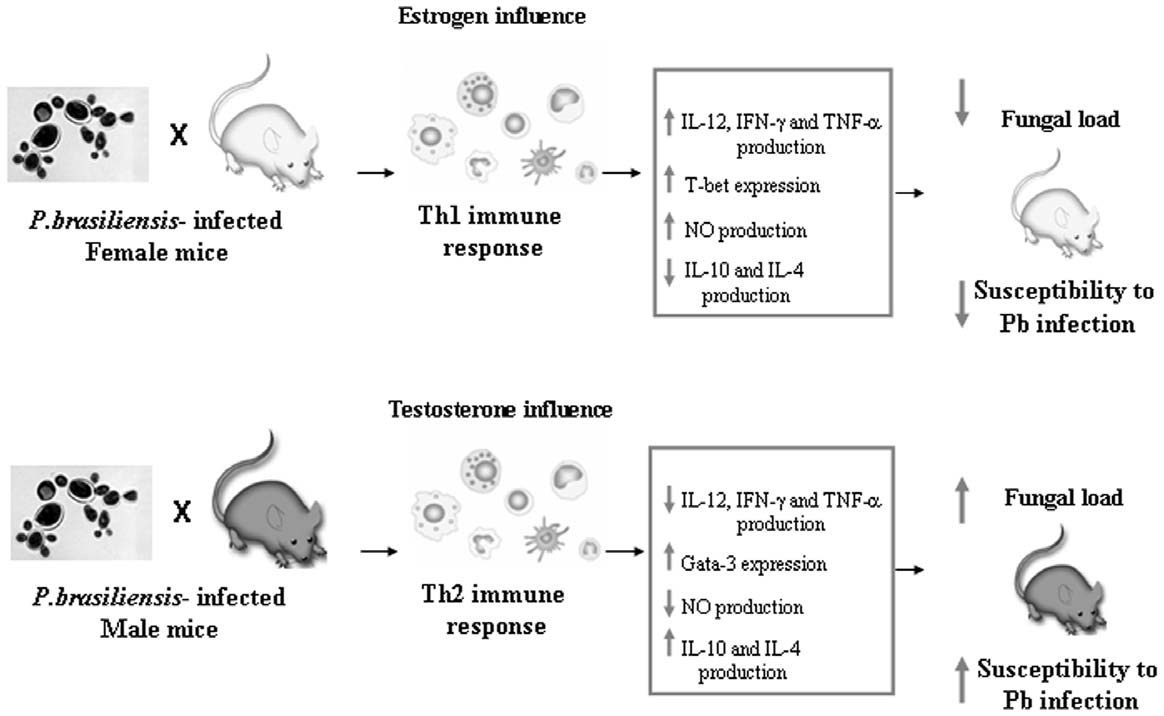
Possible role of hormones in the course of *P. brasiliensis* infection. Male and female mice were infected with *P. brasiliensis* yeasts cells and several events of the immune responses mounted by both genders of mice are displayed in this Figure.

The gender differences here outlined concern predominantly the innate mechanisms of immunity. It is clear that spleen cells from infected females soon produced higher levels of Th1 cytokines, and their peritoneal macrophages promptly reacted to yeasts by internalizing and destroying them through the cytotoxicity provided by high nitric oxide production. It is known that the innate defence events occurring in the early period of *P. brasiliensis* infection act not only to control fungal growth but also to deeply influence the adaptive response that will be developed in the subsequent period of the infection and determine the disease outcome. In this context, the production of Th1 cytokines in the early phases of *P. brasiliensis* infection is considered to drive the adaptive response toward a cell-mediated pattern that confers resistance to the fungal infection [16], [19], [36]. So the Th1/Th2 paradigm explains distinct fungal growth when susceptible *vs* resistant mice and male *vs* female mice are compared. This statement, regarding the male *vs* female mice comparison, is corroborated by our own results, which showed that spleen cells from females produced higher levels of IL-12, IFN-γ, and TNF-α. It is also consistent with the observations of Aristizabal et al. (2002), which showed a better disease outcome in females compared with males, until 6 weeks after fungal inoculation. A summary of the immunological basis for the gender differences in murine *Paracoccidioides brasiliensis* infection is shown in Figure 7.

In conclusion, our study indicates that the remarkable influence of gender on the outcome of experimental paracoccidiodomycosis is at least in part attributed to the interference exerted by sexual hormones in the immune response triggered by *P. brasiliensis* infection. In addition, the fungal antigen paracoccin, through its carbohydrate recognition property, activates innate immune cells that make female mice more resistant against *P. brasiliensis*.

## Materials and Methods

### Ethics Statement

All animals were handled in strict accordance with good animal practice as defined by the relevant national and local animal welfare bodies, and all animal work was approved by Animal Care Committee of University of São Paulo.

### Fungal isolate

The virulent *P. brasiliensis* isolate (Pb18) used in this study was cultured in a semisolid medium at 36°C for 7 days, as described elsewhere [69]. To ensure the maintenance of Pb18 virulence, this isolate was used after serial passages in C57BL/6 mice. The viability of the yeast cells, determined by fluorescein diacetate and ethidium bromide staining [70], was always higher than 90%.

### Preparation of the fungal exoantigen and paracoccin isolation

*P. brasiliensis* yeast cells were transferred from solid to liquid medium F-12 (Invitrogen Corporation, Carlsbad, CA, USA) and cultured at 36°C. After 1 week, the culture supernatant was concentrated and dialyzed against phosphate-buffered saline solution (PBS, 10 mM sodium phosphate containing 0.15 M NaCl, pH 7.2) in an ultradiafiltration system, using a YM-10 membrane (Amicon, W.R. Grace & Co., Beverly, MA, USA). The dialyzed material containing 1.0 mM phenylmethylsulfonyl fluoride (PMSF, Sigma Chemical Co., St. Louis, MO, USA) was named *P. brasiliensis* crude exoantigen (exoAg). The protein concentration was estimated by absorbance at 280 nm (Ultrospec 3000 pro, Amersham Biosciences, and Uppsala, Sweden). This crude exoantigen preparation was used to isolate paracoccin, through affinity chromatography on an N-acetyl-glucosamine (GlcNAc)-Agarose 6B (Sigma) column. The homogeneity of the preparation was analyzed by SDS-PAGE.

### Mice Infection

In this study were used 6- to 8-week-old C57BL/6 male and female mice. The mice were bred and maintained under standard conditions in the animal house of the School of Medicine of Ribeirão Preto, University of São Paulo, Ribeirão Preto, Brazil.

Mice were infected intraperitoneally (i.p.) with 5×10^6^ Pb 18 viable yeast cells in 500 µL pyrogen-free PBS and control animals received PBS only.

### Spleen cell cultures

Suspensions of spleen cells from male and female C57BL/6 mice were washed in RPMI-I (RPMI 1640 - Flow Laboratories, Inc., McLean, VA) and treated with lysing buffer (9 parts of 0.16 M ammonium chloride and 1 part of 0.17 M Tris-HCl, pH 7.5) for 4 min. The erythrocyte-free cells were then washed three times in RPMI-I, suspended in RPMI-C (containing 2 mM L-glutamine, 50 µM 2-mercaptoethanol, 100 U/ml penicillin, 100 µg/ml streptomycin [Sigma-Aldrich], and 5% heat-inactivated fetal calf serum [Hyclone, Logan, UT]), and dispensed in 24-well cell culture plates (1×10^6^ cells/well). Spleen cells were incubated in RPMI-C, either in the presence or in the absence of paracoccin (0.7 µg/ml) and LPS (1 µg/ml), and cultured at 37°C in a humidified 5% CO_2_ atmosphere. After 48h incubation, the culture supernatants were harvested by centrifugation and stored at −20°C until ELISA-based cytokine measurements.

### ELISA-based cytokine detection assay

The levels of IL-12p40, IL-4, IL-10, IFN-γ, and TNF-α in the supernatants of lung homogenate were measured by capture enzyme-linked immunosorbent assay (ELISA) with antibody pairs purchased from Pharmingen (Pharmingen, San Diego, USA). The ELISA procedure was performed according to the manufacturer's protocol. The cytokine concentrations were determined with reference to a standard curve for serial twofold dilutions of the murine recombinant cytokines.

### Culture of mouse peritoneal macrophages, phagocytosis assay and nitrite release

Groups of male and female mice were i.p. injected with 1 mL of sterile 3% sodium thioglycollate (Sigma Chemical Co). After 4 days, mice were euthanized and peritoneal cells were collected through cavity washing with 5 mL of ice-cold Hank's balanced salt solution (HBSS). Cells were immediately stored in ice, washed in HBSS, suspended in RPMI-5% medium (2×10^6^ cells/ml), and dispensed over round glass coverslips (13 mm), contained in 24-well flat bottom microtest plates. Following 4 h incubation, the nonadherent cells were gently removed, and the adherent cells were incubated in RPMI-5% with Pb18 yeasts (8×10^6^ cells/well). At time zero of incubation, purified paracoccin (50 µg/well) was added to the culture. To determine the yeast phagocitic index (PI), an average of 200 macrophages was counted and results were expressed in terms of the percent of phagocytic cells multiplied by the mean number of internalized particles [51]. In addition, macrophages from male and female mice were incubated with only paracoccin (PCN), only *P.* brasiliensis yeasts (Pb), PCN plus Pb, PCN (pre-treated with GlcNac) plus Pb or only medium. After 48 hours incubation, the cultures supernatants were collected for NO determination, by using the standard Griess reaction, in which the micromolar concentrations of NO were deduced from a standard curve using a known concentration of NaNO_2_ diluted in RPMI medium. All cultures and incubations were carried out at 37°C in a humidified 5% CO_2_ atmosphere.

### Colony forming units (CFU) determination after phagocytosis

The determination of the number of viable fungi after phagocytosis by macrophages as made by CFU counts. Macrophages from male and female mice were challenged with live Pb yeasts and incubated for 4 h, as described in phagocytic tests. After this time, cultures were rinsed with PBS for removal of non-internalized Pb cells. Distilled water was added to lyse macrophages. The cellular suspension was harvested, washed in PBS, and the final pellets ressuspended in 1 ml of PBS. Aliquots of 100 µL of each sample were plated in agar plates (4% SFB, 5% BHI solid medium). Colonies per plate were counted after 8–10 days of incubation at 37°C.

### Real Time quantitative PCR analysis

Total RNA was isolated from cells using TRIzol reagent (Invitrogen Life Technologies, Carlsbad, CA, USA), following the manufacturer's intructions. cDNA synthesis was performed in a final volume of 20 µL, using ImProm-II Reverse Transcriptase (Promega Corporation, Madison, WI, USA). The reaction mixture contained 4 µg of total RNA, 20 pmol of oligo dT primer (Invitrogen Life Technologies, Carlsbad, CA, USA), 40 U of RNAsin, 1 M of dNTP mix, and 1 U of reverse transcriptase buffer. cDNA was treated with 10 µg of RNase (Gibco, Carlsbad, CA, USA). It was then immediately used or stored at −20°C. Real-Time PCR amplification and analysis were achieved by using an ABI Prism 7500 sequence detector (Applied Biosystems). Data were normalized by β-actin gene and the relative quantification was made by the delta Ct method (Applied Biosystems, Foster City, CA, USA). All the reactions were performed with SYBR Green Master Mix (Applied Biosystems) using a 25 µL volume in each reaction, which contained 2 µL of template cDNA, 5 pmol of each primer, and 12.5 µL of SYBR Green. The primers used for PCR amplification were as follows: for IL-4, *5′- GTCTCTCGTCACTGACGGCA- 3′* (forward) and *5′- CGTGGATATGGCTCCTGGTAC -3′* (reverse); for GATA-3, *5′- AAGAAAGGCATGAAGGACGC -3′* (forward) and *5′- GTGTGCCCATTTGGACATCA -3′* (reverse);for β-actin, *5′- AGCTGCGTTTTACACCCTTT -3′* (forward) and *5′- AAGCCATGCCAATGTTGTCT -3′* (reverse); for T-bet, 5′- *CACTAAGCAAGGACGGCGAA* – 3′ (foward) and 5′- *CACCAAGACCACATCCACA* – 3′ (reverse).

### Quantification of *P. brasiliensis* by real time PCR

Molecular quantification of *P. brasiliensis* was performed as previously described [71]. Lung samples were frozen in liquid nitrogen for 30 s and pulverized. The total DNA was extracted and precipitated using the DNeasy Tissue Kit (Qiagen, Valencia, CA, USA), according to the manufacturer's protocol. PCR amplification and analysis were achieved using an ABI Prism 7500 sequence detector (Applied Biosystems, Foster City, CA, USA). Reactions were performed with TaqMan Universal PCR Master Mix (Applied Biosystems) in 20 µL solutions containing 50 ng of template DNA, 5 pmol of each primer, and probe and 10 µL of TaqMan Master Mix. Each sample was tested in duplicate and all quantifications were normalized to an endogenous control (β-actin). The primers and probes used for PCR amplification targeting the Gp43 gene [72] for *P. brasiliensis* and β-actin gene for mouse were designed using the Primer Express® software v2.0 (Applied Biosystems). The sequences for primers and probe for β-actin gene are: forward - 5′- *AGCTGCGTTTTACACCCTTT* -3′; reverse - 5′- *AAGCCATGCCAATGTTGTCT* -3′ and probe: -5-FAM-*TGACAAAACCTAACTTGCGCAGAAAAA*-Tamra-3′. The primers and probe for Gp43 gene are: forward - 5′- *FAM-GATTGATGAAGCTGCGGTTGA*-Tamra-3′ and reverse 5′- *CATACAGATCTCCGACGCTGC* -3′.

### Assay for Organ Colony-Forming Units

To assay the dissemination of the fungus to the lung and liver, the animals were euthanized after 30 days of i.p and infection and the organs were removed, weighed, homogeneized in sterile PBS (pH 7.2), and serially diluted. Aliquots of 100 µL were dispensed into Petri dishes containing brain-heart infusion agar (Difco Laboratories, Detroit, MI, USA) supplemented with 4% (v/v) of fetal bovine serum, in duplicates. Plates were incubated at 37°C, and colonies were counted 7 and 14 days later. Results are expressed as the number of colony-forming units (CFU)±SD per gram of tissue.

### Castration and Sex Hormone Treatment

The animals were anesthetized with an intraperitoneal injection of tribromoethanol (Aldrich), 0.1 ml of a 2,5% solution/10 g body weigh, and subjected to ovariectomy, orchiectomy or sham operation. In the sham-operected group an incision was made in the genital tract of animal without removal of the gonads. Animals were placed on a heating pad for 1 h to recover from the surgery, based on the protocol approved by the institutional animal care committee. Hormonal reconstitution started 30 days after surgery and continued twice per week by dissolving 50 µg/100 µL β-estradiol [1,3,5 (10)-estratriene-3,17β-diol; Sigma, St.Louis, MO] and Dihydrotestosterone (4,5α-Dihydrotestosterone; Sigma, St.Louis, MO) in sesame oil by boiling for 20 min at 150 C. This mixture (100µL) was injected subcutaneously on the back of mice. Control mice received sesame oil only.

### Statistical analysis

Statistical determinations of the difference between means of experimental groups were performed using one or two-way analysis of variance (ANOVA) followed by Bonferroni post test. Differences which provided *p*<0.05 were considered to be statistically significant.
